# National Implementation of a Group-Based Program Promoting Patient Engagement and Peer Support in the Veterans Health Administration: A Multi-Methods Evaluation

**DOI:** 10.3390/ijerph19148333

**Published:** 2022-07-07

**Authors:** Connor Drake, Melissa H. Abadi, Heather R. Batchelder, Bonnie O. Richard, Laura E. Balis, David Rychener

**Affiliations:** 1 Center of Innovation to Accelerate Discovery and Practice Transformation, Durham Veterans Affairs Health Care, Durham, NC 27705, USA; 2 Department of Population Health Sciences, Duke University School of Medicine, Durham, NC 27701, USA; 3 Pacific Institute for Research and Evaluation, Louisville, KY 40202, USA; mabadi@pire.org (M.H.A.); brichard@pire.org (B.O.R.); lbalis@pire.org (L.E.B.); 4 Department of Community Health and Family Medicine, Duke University School of Medicine, Durham, NC 27710, USA; heather.batchelder@duke.edu; 5 Whole Health Institute, Bentonville, AR 72712, USA; drychener@pire.org

**Keywords:** group program, peer-led, veterans, whole health, patient-centered care, health education, implementation, consolidated framework for implementation research

## Abstract

Evidence-based approaches promoting patient engagement and chronic illness self-management include peer support, shared decision-making, and education. Designed based on these components, Taking Charge of My Life and Health (TCMLH) is a group-based, ‘Whole Person’ care program promoting mental and physical self-care and patient empowerment. Despite evidence of effectiveness, little is known about implementation for TCMLH and similar programs. In this first-of-its-kind, multi-methods evaluation conducted between 2015–2020, we report on implementation strategies and intervention adaptations with a contextual analysis to describe TCMLH translational efforts in Veterans Health Administration (VHA) facilities across the United States. Quantitative and qualitative data were collected via listening sessions with TCMLH facilitators, open-ended survey responses from facilitators, and quarterly reports from clinical implementation sites. We used the Consolidated Framework for Implementation Research (CFIR) to analyze, interpret, and organize qualitative findings, and descriptive statistics to analyze quantitative data. Most TCMLH programs (58%) were adapted from the original format, including changes to the modality, duration, or frequency of sessions. Findings suggest these adaptations occurred in response to barriers including space, staffing constraints, and participant recruitment. Overall, findings highlight practical insights for improving the implementation of TCMLH, including recommendations for additional adaptations and tailored implementation strategies to promote its reach.

## 1. Introduction

More than half of Americans have one or more conditions that require ongoing treatment and patient self-management [1]. This is a particular challenge for veterans, who suffer from chronic conditions at a higher rate than the civilian population [2]. To improve chronic care quality and outcomes, evidence-based approaches emphasize patient-centeredness, self-management, peer support, patient education, and shared decision-making [3,4,5,6,7]. Part of the United States’ Department of Veterans’ Affairs (VA), the Veterans Health Administration (VHA) is the largest integrated health care system in the United States and has invested in the implementation of evidence-based components of high-quality chronic care through a Whole Health approach, which focuses on whole person care and includes non-clinical domains of health (e.g., social support, environment, spirituality, and personal development) [8,9]. This care paradigm differs from a reactive, disease-oriented approach by promoting the patient’s active role in healthcare planning and placing importance on the patient’s social context and priorities [10,11,12,13]. 

As part of the VHA’s overall strategic initiative to provide proactive, personalized, and patient-driven care, Taking Charge of My Life and Health (TCMLH) was developed to educate patients on self-care strategies related to their mental, physical, and emotional health, and to foster healthy behaviors through peer support. The VHA has long recognized the importance of promoting patient engagement through peer support by creating distinct position classifications designated for peer support specialists [14]. Delivered by a trained facilitator (i.e., volunteers, clinicians, administrative staff from community organizations/VHA), TCMLH leverages this organizational investment in peer support through a structured group program that provides veterans with a social environment promoting accountability in which they can (a) determine what matters most to them by exploring their life’s mission, aspirations, or purpose, (b) identify priorities for their health, and (c) acquire skills that empower them to make positive changes in their health through goal-setting [15]. Key, evidence-based instructional and interactive content includes a Whole Health self-assessment called the Personal Health Inventory (PHI) [16,17], mindful awareness practices [18], educational self-care content (e.g., nutrition, movement, stress management, and personal development) [19], and patient-driven SMART (i.e., Specific, Measurable, Action-oriented, Realistic, Timed) goal setting to promote sustainable lifestyle modifications. Veterans that have participated in TCMLH report decreased levels of perceived stress, and experience improvements in patient activation, self-care attitudes/beliefs, health goal progress, and quality of life. Feedback from stakeholders revealed the need for program adaptations to support adoption and reach [15,20]. The original curriculum was designed to be delivered via weekly, in-person sessions over 9 weeks. Adaptations that preserved fidelity to core intervention components included a telehealth version subsequently used in response to the COVID-19 pandemic [21] and abbreviated programs (e.g., six-week or two-day intensive retreats) to improve fit with existing VHA programs and practice patterns. Recent research on the transition of TCMLH to telehealth found that while challenging, the approach was feasible and offered benefits ranging from promoting access and engagement to addressing social isolation that resulted from quarantine requirements. Notably, the shift to telehealth required changes to TCMLH facilitator delivery, more robust technical support, and leveraging standardized telehealth technology in a manner that was amenable to group-based delivery and peer support [21]. Additional details related to TCMLH are published elsewhere [15,20,22]. 

There is a breadth of literature demonstrating that group-based and peer support models promote patient self-management and behavior change [4,23,24,25], and an emerging evidence base describing best practices for translating these care models into real-world settings. A 2019 systematic review of implementation barriers and facilitators of peer support interventions for mental health found that organizational culture, training, and role definition were influential to implementation [26]. Similarly, group medical visits leverage peer support within conventional clinical encounters and have unique implementation drivers including time, billing, and training [27,28,29]. There is limited implementation research (i.e., description of implementation strategies and contextual considerations) that influence implementation to promote effective chronic illness self-management outside of a traditional clinical encounter. 

Implementation science (or implementation research) can be defined as the “scientific study of methods to promote the uptake of research findings and other evidence-based practices into routine practice” with the goal of reducing the amount of time required to translate novel approaches into routine delivery. The VA has invested significantly in the field of implementation science to support more rapid movement of effective interventions into practice. For example, the Quality Enhancement Research Initiative (QUERI) is recognized as one of the oldest and most extensive scientific programs combining scientific rigor with VHA operation leaders who face implementation issues [30]. Implementation science and quality improvement efforts share common goals related to improving health care quality, but differ in focus. Quality improvement methodologies focus on overcoming challenges within a specific setting, whereas implementation science focuses on a specific practice or intervention that is underutilized, understanding its interplay with organizational context and identifying generalizable implementation strategies (i.e., discrete activities or events that influence the use of a practice or intervention). Thus, the emphasis of implementation science methods is the process of implementation and its impact on the practice or intervention of interest, often using quantitative and qualitative data derived from varying levels of observation (e.g., patients, providers, and broader environmental factors at the community or policy levels) [30]. 

To address the dearth of research describing implementation of peer support-enabled approaches, we described TCMLH implementation strategies and conducted a contextual analysis of determinants that influence activities across the spectrum of TCMLH translation into practice (e.g., adoption, implementation, and sustainability) in diverse VHA settings across the United States. Using qualitative and quantitative data from varied perspectives, our findings capture the complexity of national TCMLH implementation efforts. Our manuscript organized analyses of these data sources and findings derived from the national TCMLH implementation evaluation by means of the following: (a) presenting information on implementation strategies used to translate TCMLH into practice, (b) describing implementation context (i.e., barriers and facilitators), (c) reporting on TCMLH intervention adaptations that were made to support implementation. We organized these elements within an overarching implementation research framework to improve interpretability. By engaging key administrative and clinical stakeholders implementing TCMLH, we sought to identify opportunities for informing intervention adaptation and the refinement of implementation strategies of TCMLH as a mechanism for delivery of high-quality, patient-centered care. 

## 2. Materials and Methods

Data for this manuscript were drawn from a multi-year project to design, implement, and evaluate TCMLH (VA777-12-C-0002; Rychener, PI) throughout the VHA system from 2015–2020. Evaluation was conducted retrospectively using a multi-methods approach [31,32]. The team collected quantitative and qualitative data during the TCMLH program evaluation period (2016–2018). Data were collected from three sources: (1) listening sessions, (2) open-ended survey responses, and (3) quarterly site implementation reports.

### 2.1. Data Collection and Measures

#### 2.1.1. Listening Session Qualitative Data Collection

Listening sessions lasted 40–45 min and were conducted with the first 8–10 participants who signed up at the conclusion of each of six facilitator training courses to gain in-depth feedback on trainees’ perspectives. The listening session guide (Supplementary Materials) explored aspects of TCMLH adoption and implementation including: (i) their perceptions about the TCMLH group program’s value to veterans; (ii) anticipated challenges with TCMLH implementation at their VHA sites; and (iii) details on their plans for facilitating TCMLH programs. Listening sessions were audio-recorded and transcribed, with identifying information removed. 

#### 2.1.2. Survey Qualitative Data Collection

Data on facilitators’ implementation experiences were collected through two facilitator open-ended questions on a larger follow-up survey. The survey was emailed to facilitators to complete online within two to four weeks following completion of TCMLH program delivery. Surveys were anonymous and took approximately 10–15 min to complete. To assess experiences related to TCMLH program implementation, we asked: (1) What are some challenges you faced in facilitating group sessions with veterans to discuss self-care strategies for better health and emotional well-being? and (2) What, if anything, would you like to see done differently in terms of recruiting veterans for group programs at your facility [22]? 

#### 2.1.3. Site Implementation Report Data Collection

Site implementation reports were collected quarterly between 2016–2018 from eight VA sites/systems offering the TCMLH program. These sites were based in the South, Southeast, Mid-Atlantic, and Midwest of the continental United States. Site point of contacts (POCs) were the primary liaisons between the implementation sites and the evaluation team. POCs, with consultation from front-line staff and clinicians, provided information on TCMLH programs and adaptations. Information included start dates, format (e.g., in-person, telehealth, or combined), characteristics of each group (e.g., number of attendees), time period duration (e.g., 9-week, 6-week, 4-h, 2-day, or other), number of sessions, facilitator characteristics (e.g., clinician, staff, or volunteer), open-ended questions regarding recruitment processes (i.e., type of strategies used, strategies that “worked well”, and strategies that “did not work well”) and implementation challenges. POCs were asked to provide suggestions for future recruitment, as well as for future TCMLH implementation. POCs completed the site reports using an online survey or by email. When necessary, evaluation team members contacted POCs via email or phone to follow-up on missing data or clarify details. 

#### 2.1.4. Implementation Strategies

**Figure 1:**
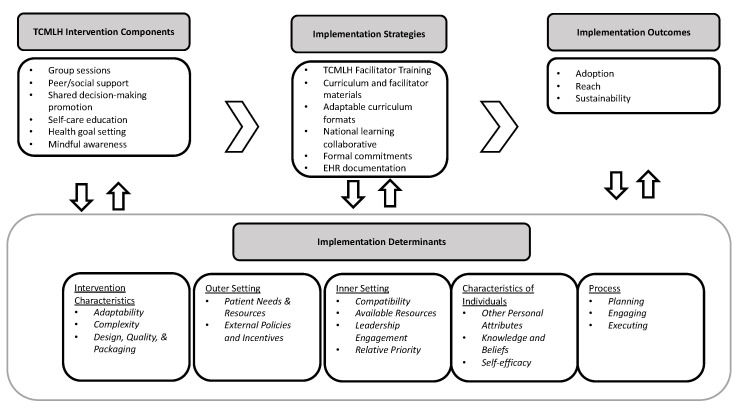
TCMLH Intervention and Implementation Logic Model.

To support the translation of TCMLH into routine practice patterns, implementation strategies, defined as the “methods of techniques used to enhance the adoption, implementation, and sustainability of a clinical program or practice [32]”, were used to support translational efforts in participating VHA sites. TCMLH sites were provided with a combination of implementation strategies, including the aforementioned facilitator training, program materials, and technical assistance. See Table 1 for additional description on the strategies used, consistent with the Expert Recommendation for Implementing Change (ERIC) specifying and reporting criteria [33,34]. Figure 1 is a model, or depiction of assumed relationships commonly presented in implementation research [30] that illustrates the dynamic interplay of TCMLH intervention characteristics, implementation strategies, and contextual factors, that hinder or promote adoption, implementation, and sustainability.

All protocols were approved by the Pacific Institute for Research and Evaluation (PIRE) Institutional Review Board (IRB) and deemed exempt due to the low risk of the evaluation. In addition, the VHA’s Office of Patient Centered Care and Cultural Transformation classified this evaluation as a part of the ongoing development of the VHA’s Whole Health System Quality Improvement Initiative.

### 2.2. Data Analysis

The Consolidated Framework for Implementation Research (CFIR) was selected as an implementation research framework to inform analysis and the organization of results. CFIR can be used to assess and identify potential barriers and facilitators to implementation and inform tailored implementation strategies to promote the uptake of an intervention into practice [35]. The five CFIR domains (*Intervention Characteristics, Outer Setting, Inner Setting, Characteristics of Individuals*, and *Process*) and corresponding nested constructs, or more granular sub-categories, that are associated with the domain, based on existing literature and theory, were used to identify and organize TCMLH implementation determinants.

#### 2.2.1. Listening Session Qualitative Data Analysis

Qualitative data were analyzed using a grounded approach wherein concepts and ideas expressed by participants were grouped together under broad topical themes, and exemplary quoted statements were then selected to represent common ideas [36]. Representative quotations were extracted across these themes, and findings were included in a report as a data reduction analysis [37]. 

Two trained qualitative researchers (CD and HB) used a directed content analysis approach to apply CFIR domains and nested constructs to the exemplar quotes in the report. This process involved developing a codebook a priori from CFIR domains and constructs. The two coders independently reviewed responses and coded them using the codebook. This led to the refinement and update of the initial codebook to inform a consistent coding methodology. Once the codebook was finalized, all responses were reanalyzed for the final codebook, and coders met regularly to ensure codes were consistently applied. Differences between coders in application of CFIR domains and constructs were reconciled through an iterative process and exemplary quotations were identified. 

#### 2.2.2. Survey Qualitative Data Analysis

For qualitative data from facilitators’ follow-up surveys, two trained coders (CD and HB) used a directed content analysis approach (see the aforementioned description of the approach) to analyze responses to open-ended implementation questions [38]. When necessary, a separate coder (BR) was involved in discussions to help clarify responses and assist in resolving coding differences. 

#### 2.2.3. Site Implementation Report Data Analysis

Site implementation reports contained a combination of qualitative (i.e., open-ended survey response questions) and quantitative data (i.e., ordinal, or numeric responses). Quantitative data, including attendance, number of sessions, and facilitator characteristics, were reported by frequency and, when applicable, descriptive statistics were used for analysis. Qualitative data, including responses to open-ended questions on methods of recruitment, methods that worked well and those that did not, and program adaptations, were summarized, based on thematic frequency of responses by an experienced qualitative researcher (BR).

## 3. Results

### 3.1. Participants

#### 3.1.1. Sample for Listening Sessions

Listening sessions had a mean of 8.66 participants across 6 sessions. A total of 52 out of 97 facilitator training attendees participated. Participants were a voluntary, self-selected subsample.

#### 3.1.2. Sample for Facilitator Survey

The response rate from the 75 trained facilitators representing 41 VHA health systems regarding completion of the post-TCMLH implementation survey was 93%. Overall, respondents were veterans (67.1%) and White, non-Hispanic (51.4%). Occupations included peer support specialist (28.6%), administrative staff (24.3%), registered nurse or nurse practitioner (15.7%), volunteer (12.9%), and social worker (8.6%). Additional demographic characteristics are presented in Table 2. 

#### 3.1.3. Sample for Site Implementation Reports

Across all sites, 133 TCMLH programs were completed between fiscal years 2016–2018 in diverse geographic regions and community types (e.g., urban, suburban, rural). These programs were delivered by a mixture of volunteers (n = 10) and VA staff (n = 71) facilitators. Additional details can be found in Table 3.

### 3.2. Evaluation Findings

Across the listening sessions, open-ended survey responses, and site implementation reports, POCs and TCMLH facilitators described various aspects of the TCMLH implementation, noting possible implementation facilitators, barriers, and opportunities. Additional details on CFIR domains and corresponding constructs, descriptions, and illustrative quotes are in Table 4. CFIR domains and nested constructs are italicized.

#### 3.2.1. Intervention Characteristics

TCMLH’s *Intervention Characteristics*, or the attributes of the program that influence implementation success, were a determinant of the ability to introduce the program as a clinical wrap-around service according to respondents across all three data sources. POCs and facilitators commented on how the TCMLH program’s *Design Quality and Packaging* was an important consideration for implementation, as was how TCMLH is presented to both clinicians and veterans (e.g., the description, purpose, packaging, and accompanying materials of the program). In open-ended survey responses, respondents noted that covering all the curriculum content, as well as covering missed material when Veterans missed sessions, was difficult. In listening sessions, facilitators communicated a desire for more comprehensive and extensive training on the use of TCMLH intervention materials, including the facilitator manual and participant workbook. 

Open-ended survey responses suggested that TCMLH should include more options for adapting the duration, recommended group size, delivery modality (e.g., in-person versus utilizing telehealth), and target population of the program. One facilitator noted,

“The number of sessions seems to be a barrier. Many don’t want to commit to 9 weeks. Adapting program to be of shorter duration might attract more interest”.

To this point, the overall *Adaptability*, or the degree to which TCMLH could be tailored, refined, or reinvented to meet local needs, was reported to promote adoption and sustainability. POCs detailed executed adaptations to the TCMLH program to meet local operational and population needs, including changing the duration (ranging from a two-day weekend format up to 12 weeks) and delivery modality (e.g., in-person, telehealth, or hybrid). Over fifty percent of TCMLH programs were adapted from the original 9-week format, and 10% were adapted to incorporate telehealth (see Table 5 for additional details on adaptations and attendance). The rationale for these adaptations was related to scheduling preferences of staff and veterans in order to improve programmatic access. The most abbreviated version of TCMLH, a 2-day intensive “weekend retreat” format, had a higher attendance rate than TCMLH delivery that occurred over 8, 9, and 12 weeks. Finally, in open-ended survey responses, facilitators suggested that the *Complexity*, or perceived difficulty or intricacy, of TCMLH may hinder implementation. Specifically, facilitators mentioned the inherent complexities of delivering a group-based program, including factors such as time and group management, were especially difficult in a telehealth context. Related to the challenges of group management, in an open-ended survey response a facilitator commented:

“When in a group setting, conversations often turn to advice giving which is sometimes difficult to redirect. It is also difficult to make sure everyone’s concerns are addressed while not allowing one person to dominate the conversation”.

#### 3.2.2. Outer Setting

Qualitative data from facilitator surveys and listening sessions described CFIR constructs related to the *Outer Setting*, or the external systems that influence how the intervention is adopted or sustained. Findings indicated that *Patient Needs and Resources* influenced implementation. Specifically, several facilitators reported in open ended survey responses that challenges arose from participants not being comfortable articulating their health goals or discussing sensitive topics during group visits. One facilitator noted that not all veterans were “…comfortable sharing personal health and wellness challenges with peers,” whereas another noted that, “there are some areas (e.g., sex) [during the program] that male veterans are not all that comfortable talking about with a female and vice-versa”. Facilitators also noted in open ended survey responses that certain veteran attributes impacted program delivery and success. Specifically, veterans’ varying motivations and readiness for change, differences in communication styles, and disruptive behaviors could shape or impede TCMLH delivery. Facilitators indicated in open-ended survey responses that some patients tended to “dominate conversations, taking over the content which needs to be delivered” and those with “strong personalities” could create challenges with group dynamics and management. Facilitators suggested future groups improve their screening process so that those enrolled were participants who were ready to participate in a supportive group setting and had sufficient levels of motivation and readiness to engage with the program.

*External Policies and Incentives*, or the policies and regulations that influence implementation, including performance measurement, incentives, and benchmark reporting, were a consideration for TCMLH adoption, implementation, and sustainability. Specifically, a facilitator in an open-ended survey response highlighted the importance of logistical considerations surrounding applying the correct stop codes (i.e., quality reporting) when setting up clinics in the electronic health record and documenting of TCMLH delivery.

#### 3.2.3. Inner Setting

Findings from both qualitative data sources described several CFIR constructs related to the *Inner Setting*, or internal facets of the intervention’s interaction with the implementation setting. Some of the constructs led to successful implementation of the TCMLH program, whereas others were identified as areas for improvement in future iterations of the TCMLH program. 

*Compatibility* of the TCMLH program with VHA organizational priorities, goals, and values was described as a factor that helped promote adoption and sustainability. In listening sessions, facilitators believed that the material would be effective in helping veterans improve their health and that the program was aligned with the VHA mission and organizational priorities to provide comprehensive, patient centered care. One facilitator noted,

“I think it will hopefully give another perspective in which [Veterans] can look at their health...finding connections between all these different aspects of their lives that they may not see”.

Additionally, in listening sessions, facilitators commented that the program would support self-care skill development versus sole reliance on medical treatment and pharmacological intervention to help manage chronic conditions, such as chronic pain. Some described this approach as present within other programmatic areas and initiatives within the health system and expressed that the TCMLH program aligned with this shift towards a Whole Health approach, which is compatible with the VHA system’s goal of a more comprehensive approach to care and promoting patient self-care and empowerment.

A recurring theme reported across all three data sources was related to *Available Resources* or organizational capacity for TCMLH implementation and sustainability. This included physical space, room reservations, and administrative support for recruitment efforts. These resources were often difficult to obtain and maintain. One barrier to implementation concerned the physical space for holding group programs. For POCs, physical space to hold the programs was limited and they were not large enough to support the size of groups. Further, the process to reserve the same room for all sessions was complicated. In addition, POCs at several sites described that they could only schedule programs during weekday business hours, thus hindering attendance from veterans still in the workforce. Finally, POCs noted that participant recruitment was time-intensive and lacked sufficient administrative coordination. Specifically, more staff effort and organizational infrastructure to adequately support recruitment efforts (e.g., develop referral pathways and advertising campaigns) for the program was needed. Facilitators commented that the use of creative marketing angles and advertising materials, as well as simplified referral mechanisms to facilitate provider and care team integration, could enhance the reach of TCMLH.

Both POCs and facilitators felt that *Leadership Engagement* and organizational *Relative Priority* were key implementation drivers. Facilitators reported, via open ended survey responses and listening sessions, that TCMLH implementation guidance from site leadership was inconsistent and that they were often without clear direction. They also expressed that there was a lack of follow-through from leadership in scheduling training and programs, and that leadership did not clearly identify ways to sustain operation of TCMLH. POCs described a dearth of programmatic support and capacity to allow facilitators to be adequately available to facilitate the groups. For example, one listening session respondent indicated,

“I’m looking at it from the programmatic or organizational point of view... without some clear accountability, without giving it importance from the leadership level, how do I pull her as an asset when I need her [to facilitate a group] when she’s supposed to be doing x, y, and z?”

In both listening sessions and open-ended survey responses, facilitators expressed that there needed to be more buy-in from specialty clinics and providers, suggesting that systemwide uptake of the Whole Health approach would better align VHA health care with what veterans learn through the TCMLH program. They elucidated that participants would learn about the holistic approach to health management through the TCMLH program, but the VHA clinics, from which participants sought medical care, were not always aware of the program and were not able to reinforce self-care priorities identified through TCMLH participation. Similarly, open ended survey responses suggested that clinicians and community partners should receive more education on the goals of the program. In a listening session, a facilitator summarized these concerns related to the complex organizational dynamics, priorities, and norms within a health care system that complicates efforts to implement TCMLH as part of a new, Whole Health care paradigm,

“By nature, we’re resistant to change.... whenever I heard the word, culture transformation, I know that’s going to be a fight.... Because you are going to face resistance until you get to buy-in and everybody gets in on different levels, and some people will never buy into what is happening.... and you have to have that fortitude to fight through that change. But I see this is what exactly what is needed. Being a recent Veteran and still connected to those who serve, I tell you, this is needed”.

#### 3.2.4. Characteristics of Individuals

The skills, training, and motivations of the implementing personnel, or the *Characteristics of the Individual*, were described as significant drivers of implementation. Described under the *Other Personal Attributes* nested construct, several facilitators described group engagement and active listening and reflection as key skills required to implement TCMLH that were enhanced through participation in the TCMLH facilitator training. One facilitator remarked,

“Reflection, paraphrasing— I feel like I was familiar with these concepts in theory, like somewhere I’ve heard them before, but I wasn’t sure I was buying into them.... I think practicing it and seeing how it works in action was really what made me a believer”.

The training was complemented by real-world experience that helped enhance this skillset and allowed them to, as one facilitator described in an open-ended survey response, “trust the process” and “let the group dynamic flow”. However, group management and patient engagement were described as challenging skills that were made more difficult by the diverse range of participant backgrounds, personalities, and health conditions. *Other Personal Attributes*, which includes attributes that the facilitator may or may not have to successfully engage participants, influenced implementation. For example, in open-ended survey responses one facilitator voiced that they felt unsure about how to encourage consistent veteran participation in the TCMLH program, “Ensuring individual participation is always a challenge…” Another commented that “with anything new, it takes time. Gaining the trust from the veterans and allowing them [to] trust the process”. 

Facilitators in listening sessions also shared their *Knowledge and Beliefs about the Intervention*. Specifically, facilitators reported that they felt more hopeful and enthusiastic about the positive impact the TCMLH program would have on the VHA system and veterans’ health. One facilitator articulated a common perception of TCMLH benefitting veterans, “I think our outcomes will be better, I think our patient satisfaction score is going to go up…”. Additionally, there were facilitators that reported that their own beliefs in their capabilities to execute the program, or *Self-efficacy*, could advance implementation goals without advanced clinical training. In a listening session, a facilitator summarized this theme by saying, “It’s like, alright, this is something we can really roll out for the Veterans and we don’t have to be PhDs or doctors”.

#### 3.2.5. Process

POCs and facilitators included descriptions of TCMLH implementation activities related to the *Process* domain and *Executing* construct. The most notable of these areas involved recruitment of participants into the program. As part of TCMLH implementation, sites planned and utilized various methods of recruitment, including clinician referrals/consults, outreach via program representatives, introducing the program during veteran orientation events, passive media advertising (e.g., flyers), promoting TCMLH at other VHA group programs, follow-up/reminder calls, and word of mouth from other veterans. As reported in the site implementation reports (See Table 6), the most frequently used and highest rated modalities for recruitment were the following: (a) direct outreach to patients by TCMLH program representatives (used by 7/8 sites with 6/7 reporting that the method “Worked Well”), and (b) introduction of the TCMLH program during new patient orientation events (used by 6/8 sites with 5/6 reporting that the method “Worked Well”). Clinician referrals and passive media advertising (e.g., flyers) were also used by most sites but with less success (50% reported that the method “Did Not Work Well”).

Designing effective recruitment methods required consideration and resource inputs related to *Planning*. POCs responded that recruitment efforts could have been executed more successfully with more effort in allocation of administrative tasks and more organization from program leadership. Facilitators also reported, via open-ended survey responses and listening session responses, that recruitment needed to include efforts from additional staff to recruit/refer patients to the program. For example, a facilitator suggested that future implementation would be improved if there was “an avenue for providers to refer patients directly to our group”. Other facilitators commented on how diversifying staff or clinics that recruited for TCMLH would improve overall recruitment capacity, by allowing “facilitators to participate in recruiting,” or “having the CBOC (community-based outpatient clinic) refer clients to the group”. 

Implementation efforts related to *Engaging* involved attracting appropriate individuals in the implementation and use of the intervention through social marketing and other outreach efforts. One facilitator noted that this was a persistent challenge within the VA due to lack of awareness of available VA programs and services,

“I think [a challenge will be] getting the word out to Veterans it’s available to them and how helpful it can be. I see constantly that Veterans that have no clue what services and supports are available to them”.

Facilitators and POCs commented on activities, strategies, and approaches that could improve awareness and reach of the TCMLH program. For example, facilitators in open ended survey responses suggested allowing spouses/partners to participate and to recruit veterans from other clinics or community-based organizations that could benefit from the program. To do so, multiple facilitators suggested, in open-ended survey responses, the use of more engaging flyers/visual aids and posting them in high traffic locations like the “[VA] entrance and by the pharmacy and emergency room areas,” or on television monitors within the VA. POCs suggested utilizing existing VHA systems (i.e., Office of Public Affairs) to enhance awareness system-wide and strategically timing recruitment methods to allow for consistent messaging and delivery. 

Facilitators described *Executing* considerations related to program delivery that ranged from telehealth delivery considerations, optimal group sizes, and techniques for engaging Veterans to participate fully. For example, in a listening session one respondent indicated that, “When [your] group is 12 or over it is hard to cover all the information”. In a similar vein, facilitators noted that the nuanced curriculum of the program complicated efforts to deliver content in a timely manner. One facilitator commented in an open-ended survey response,

“Being asked questions, and trying to answer and stay on the course without too much time spent on the question. Some very good and pertinent questions, I must admit”.

## 4. Discussion

The aim of this study was to conduct a contextual analysis of a multi-year national implementation of TCMLH to better understand the factors that influenced implementation and could ultimately promote intervention adoption in an effective and sustainable manner. The results of this evaluation will be used to further adapt and improve TCMLH. Similar to previous evaluations of comparable group-based interventions for health promotion in the VHA (e.g., MOVE! [39], Diabetes Prevention Program [40]), we used CFIR to analyze data and organize implementation factors. Experiences from diverse clinical settings and stakeholder perspectives are critical to inform translation of TCMLH, and programs like it, into routine practice. To this end, several important findings emerged. 

### 4.1. The Interplay between TCMLH Adaptation and Implementation

Site autonomy to adapt the TCMLH program might have supported implementation efforts. Intervention adaptation in ‘real-world’ settings is a topic of considerable interest in the field [41]. Intervention adaptations included: changes to session and program length, incorporation of telehealth technologies, and facilitator staffing models. We found that the majority of TCMLH programs (58%) were adapted from the original length and a portion deviated from in-person delivery to using telehealth (10%) pre-COVID. Given that it is unlikely that the same program, techniques, and strategies can be implemented in the exact same way across multiple settings, adaptation is inevitable during the implementation process [42,43,44]. Understanding the rationale for adaptations (e.g., cultural or to improve contextual fit) can improve replication and translational efforts [45,46]. This study contributes by highlighting how contextual factors at multiple levels shape adaptation to continue to move away from a “one-size-fits-all” model of implementation. Specifically, qualitative findings suggested that these adaptations were in response to reported organizational barriers and facilitators. For example, lack of space for group sessions and barriers to participant recruitment and retention may have justified use of telehealth modalities and a reduction in the length of the program. Adaptations to TCMLH were both planned (e.g., changes in duration) and unplanned (e.g., delivery modality using telehealth) and occurred by means of local implementers, with direction from program developers on the core components of TCMLH that should be preserved (e.g., self-assessment using the PHI, mindful awareness, SMART goal setting, and self-care educational topic areas) [15]. As a result, the local adaptations described were consistent with Stirman et al.’s framework [44,46] for classifying “contextual modifications” (e.g., changes to format, setting) without altering content of the intervention. Even TCMLH programs that were offered over shorter durations retained the same core curriculum features. However, future research should evaluate the comparative effectiveness of adapted TCMLH programs by modality type. Relevant to the adaptation to a telehealth format observed in this study, there is preliminary research on a similar chronic illness prevention program that suggested that adapting the program to telehealth did not diminish effectiveness [47,48]. 

### 4.2. Organiztional Alignment and Beliefs about TCMLH

TCMLH implementation benefitted from alignment with the VHA’s organizational mission and the belief that the program would improve Veteran health outcomes. The description of TCMLH’s alignment with the VHA organizational priorities and mission may be due to the emphasis on providing veterans with “personalized, proactive, patient-driven health care”, as described in the VHA’s strategic plan between 2013–2018 [49]. While the shift towards a Whole Health care paradigm extends beyond one program or clinical offering, this study provides insights into how TCMLH could be a vehicle for enabling the type of patient engagement required in a delivery paradigm rooted in shared decision making. 

### 4.3. Resource Availability and Organizational Capacity

Resource availability and organizational commitment to TCMLH was described as a major driver of implementation. Lack of capacity and resources associated with space, time, referral, retention, and recruitment infrastructure/processes, and personnel availability represented barriers to TCMLH implementation. Specifically, we found that reach and awareness of TCMLH were hindered by personnel constraints related to scheduling, as well as a lack of a centralized media campaign or strategy, and lack of recruitment and referral pathways. These findings suggest that future research should select new, and adapt existing, implementation strategies that could leverage facilitators and overcome barriers. These implementation strategies should be compiled using rigorous, stakeholder-engaged methods [50] and should be tested. For example, a TCMLH implementation toolkit could include patient- and clinician-facing advertising materials and media campaigns, suggested referral mechanisms and recruitment strategies, practice facilitation and guidance on adapting the program, clinical decision support, workforce development modules, and technical assistance for incorporating telehealth technologies when physical space is unavailable. This work would build on encouraging results from the existing TCMLH facilitator training [22], which supported implementation efforts by equipping facilitators with the skills, techniques, and experiential training to deliver the TCMLH program despite its complexity and novelty. 

### 4.4. Limitations

The findings of this study should be interpreted through the lens of several limitations. The units of analysis were site POCs and facilitators. TCMLH participant perspectives specific to implementation challenges and facilitators were not included. Another limitation was that data collection instruments were not designed using CFIR *a priori* which limited the precision by which implementation context could be measured. However, data collection strategies and measures had a strong emphasis on implementation. Additionally, participation among facilitators and clinical sites was voluntary and, therefore, subject to self-selection bias. Finally, while we took steps to encourage objective responses (e.g., keeping responses confidential and aggregating site information), there may have been social desirability bias given the subject matter and nature of the relationship between respondents (e.g., site POCs and facilitators) that may have influenced the implementation process. Similarly, risk of bias on the part of the researchers that analyzed qualitative data was present, but mitigated by a process that involved multiple, experienced qualitative researchers (CD, HB, and BR) using best practices for reconciling conflicts and reducing bias. 

### 4.5. Future Research Directions

Despite these limitations, this implementation evaluation of TCMLH is the first of its kind and leverages a unique program evaluation dataset over a three-year period in diverse settings. The richness of the quantitative and qualitative data provides unique insights into implementation of TCMLH. This study illustrates several important directions for future research. Most notably, the contextual factors that influenced implementation are important inputs to refining implementation strategies of TCMLH and programs like it. By reporting the implementation strategies used and highlighting barriers and facilitators to implementation, practitioners and researchers can design de novo strategies or modify existing strategies to enhance implementation support. For example, our findings provide insights into organizational capacity building activities and recruitment strategies that could extend the reach of TCMLH. Also, while improvements in attendance were observed in shortened TCMLH programs, we cannot draw any causal inferences on this relationship. Additional research is required to determine whether this adaptation can enhance reach and retention while, as alluded to previously, not compromising effectiveness. Finally, future research should build off this work through an implementation-effectiveness trial [51] to simultaneously test the impact of TCMLH on relevant outcomes of interest and the impact of expanded implementation strategies.

## 5. Conclusions

Patients working to prevent and manage chronic conditions require patient-centered programs and clinical interventions that provide accountability, peer support, and self-care. The TCMLH program in the VHA and programs like it advance these aims and require careful attention to implementation. Our three-year program evaluation across eight sites reports implementation determinants from clinical and administrative stakeholders using CFIR. We reported the implementation strategies used to promote TCMLH adoption, implementation, and sustainability. Further, we identified a wide array of contextual factors, including resource availability, characteristics of individuals implementing TCMLH, and the needs of patients, that have practical implications for program adaptation and delivery. To this end, our findings highlight how intervention adaptations can move implementation efforts away from a ‘one-size-fits-all’ model towards a collaborative, tailored problem-solving approach that can overcome barriers related to organizational capacity, while preserving fidelity to core TCMLH intervention components. Further, these findings highlight the need for the refinement of existing implementation strategies and addition of novel strategies to improve TCMLH implementation effectiveness. We organized and presented approaches to participant referral and recruitment, service line integration, and leadership engagement from frontline stakeholders that could complement existing implementation strategies to accelerate the use of TCMLH and peer support enabled self-care interventions like TCMLH as part of routine practice patterns. By harnessing the lessons learned from this multi-year, national implementation evaluation, embedded implementation researchers and practitioners can partner up to ensure that strategies used to improve implementation are appropriately matched to the unique context in which a novel approach to peer support, like TCMLH, is introduced. 

## Figures and Tables

**Table 1:** Reporting of TCMLH Implementation Strategies Using the Expert Recommendations for Implementing Change (ERIC).

TCMLH Implementation Strategy	Description ^1^	Associated *ERIC Discrete Implementation Strategy* and Definition ^1^
TCMLH Facilitator Training	The Whole Health Facilitator Training Course is a 3-day course provided to Veteran volunteers and VHA staff to prepare them to facilitate TCMLH. A combination of didactic and experiential learning formats provides participants with knowledge of the curriculum and program scope as a non-clinical wellness resource, as well as with opportunities to practice facilitation skills, group management skills, active listening skills, and program delivery [22]. Training participants also had the opportunities to practice using the Participant Manual and Facilitator Guide. The training course promoted both skills associated with intervention delivery and skills associated with implementation, including logistics, raising awareness, and engaging leadership to promote efforts to adopt TCMLH across service lines in a sustainable manner.	*Make the training dynamic* entails varying information delivery methods to cater to different learning styles and work contexts and to shape the training innovatively to be interactive. *Recruit, designate, and train for leadership* includes the recruitment, designation, and training of organizational leaders for the change effort. *Identify and prepare champions* is the process of identifying and preparing individuals who dedicate themselves to supporting, marketing, and driving through implementation.
TCMLH Curriculum and Facilitator Materials	TCMLH intervention materials included the facilitator guide and participant manual, available in print form. The Facilitator Guide included scripts for each session, Whole Health tools, worksheets, and resources. The Participant Manual included outlines for each session, Whole Health tools, worksheets, and resources. Facilitators also had access to a DVD with some of the videos suggested in the curriculum.	*Develop educational materials* includes format manuals, toolkits, and other supporting materials being developed in ways that make it easier for stakeholders to learn about the innovation and for clinicians to learn how to deliver the clinical innovation. *Distribute educational materials* refers to the process of providing guidelines, manuals, and toolkits in person, by mail, and/or electronically.
Adaptable TCMLH Curriculum Formats	In response to stakeholder feedback, adaptation guidance was provided to preserve TCMLH core components in various formats, including 9-week, 6-week, and 1–2 sessions with various follow-ups. This guide was included in the Facilitator Manual and also provided and discussed in Community of Practice learning collaborative meetings.	*Promote adaptability* involves identifying the ways clinical innovation can be tailored to meet local needs and clarifying which elements of the innovation must be maintained to preserve fidelity.
A National Learning Collaborative	Monthly Community of Practice calls were available to all trained facilitators to provide real-world support and guidance from peers in the field also implementing TCMLH groups. Sites would present implementation and program delivery challenges and discuss solutions.	*Create a learning collaborative* is the formation of groups of providers or provider organizations and fosters a collaborative learning environment to improve implementation of clinical innovation. *Capture and share local knowledge* is the process of sharing knowledge on implementation with other sites
Formal commitments of support for implementing TCMLH	TCMLH facilitators needed commitments from their direct supervisors to protect time dedicated to implement and facilitate TCMLH within their scope of duties. This commitment was required to be in writing before a prospective facilitator could receive training.	*Obtain formal commitments* includes written commitments from key partners that state what they will do to implement the innovation.
Electronic health record documentation for quality measurement and financial incentives	TCMLH group encounters were coded for performance reporting and additional reimbursement through available financing mechanisms to promote adoption of ‘Whole Health’ programming.	*Develop and organize quality and monitoring systems* involves procedures that monitor clinical processes and/or outcomes for the purpose of quality assurance and improvement.

^1^ Descriptions use Proctor et al.’s recommendations for specifying and reporting implementation strategies across dimensions including: the actor, action, action targets, temporality and dose.

**Table 2:** Demographics and Characteristics of TCMLH Facilitator Survey Respondents (n = 70).

Demographics and Characteristics	n (%)
Female	34 (48.6)
Age-mean (min-max)	47.6 (29–74)
Veteran	47 (67.1)
**Race and Ethnicity**	-
Hispanic/Latino	7 (10.0)
Black, non-Hispanic	25 (35.7)
White, non-Hispanic	36 (51.4)
Asian	2 (2.9)
Other/missing	5 (7.1)
**Education**	
High school	2 (2.9)
Some college	15 (21.4)
College degree	17 (24.3)
Some graduate	23 (32.9)
Graduate degree	13 (18.6)
**Occupation**	
Peer support specialist	20 (28.6)
Health coach	4 (5.7)
Whole Health coordinator or partner ^1^	2 (2.9)
Social worker	6 (8.6)
Nurse (RN or NP)	11 (15.7)
Physician	1 (1.4)
Volunteer	9 (12.9)
Administrative staff	17 (24.3)

^1^ Position designated for expansion and support of Whole Health program offerings at the clinical site.

**Table 3:** Description and TCMLH Staffing by Site, FY2016-2018 Quarterly Site Reports.

VHA Site	Geographic Region	Community Type	No. of Volunteers Facilitating	No. of Staff Facilitating	Total No. of Facilitators	No. of Programs with a Volunteer Facilitator	No. of Programs Completed
Site 1	Midwest	Urban	1	6	7	2	10
Site 2	Midwest	Urban	0	3	3	0	8
Site 3	Midwest	Suburban	3	12	15	7	23
Site 4	Southeast	Suburban	0	4	4	0	4
Site 5	South	Rural	4	22	26	13	27
Site 6	Midwest	Urban	0	2	2	0	5
Site 7	Midwest	Suburban	1	14	15	1	44
Site 8	Mid Atlantic	Urban	1	8	9	3	12
**Total**			10	71	81	26	133

**Table 4:** CFIR Domains and Nested Constructs Related to TCMLH Program Implementation.

Domain Description ^1^	Nested Constructs ^1^	Illustrative Quote from Open-Ended Survey Responses and Listening Sessions
*Intervention Characteristics* refers to the key attributes or components of the intervention.	*Adaptability*, or “the degree to which an intervention can be modified or refined to meet local needs and context”.	“…The number of sessions seems to be a barrier. Many don’t want to commit to 9 weeks. Adapting program to be of shorter duration might attract more interest. Most staff not buying in to whole health and incorporating these principles into their practice”. (Facilitator feedback via open-ended survey response)
	*Complexity*, or the “perceived difficulty of the intervention, reflected by duration, scope, radicalness, disruptiveness, centrality, and intricacy and number of steps required to implement”.	“The administering of the PHI was done with too much frequency. Veterans often complained about filling this scale too many times”.—Facilitator open-ended survey response”. (Facilitator feedback via open-ended survey response)
	*Design Quality and Packaging*, or “perceived excellence in how the intervention is bundled, presented, and assembled”.	“Time—there was far more in the curriculum than we could cover”. (Facilitator feedback via open-ended survey response) “Firm mission statement. More creative learning—hands on projects to spark new thinking about self”. (Facilitator feedback via open-ended survey response) “When your group is 12 or over it is hard to cover all the information”. (Facilitator feedback via open-ended survey response)
*Outer Setting* refers to external interacting attributes or components influencing the intervention	*Patient Needs and Resources*, or “the extent to which patient needs, as well as barriers and facilitators to meet those needs, are accurately known and prioritized by the organization”.	“…some Veterans are not really open to self-care because they are use[d] to doing as told or being a provider so they tend to put themselves last based off programming”. (Facilitator feedback via open-ended survey response) “Some Veterans did not feel comfortable sharing their experiences”. (Facilitator feedback via open-ended survey response)
	*External Policies and Incentives* is “a broad construct that includes policy or regulations, external mandates, recommendations and guidelines, pay-for-performance, and benchmark reporting”.	“…Starting groups in other clinics. Logistical arrangements (e.g., setting up clinics, checking stop codes, schedule, making modifications to group to suit needs of specialty programs)”. (Facilitator feedback via open-ended survey response)
*Inner Setting* refers to the internal active interacting facets of the intervention	*Compatibility*, or “the degree of tangible fit between meaning and values attached to the intervention by involved individuals, and how the intervention fits with existing workflows and systems”.	“I’m walking away with a different view, now I’m seeing that the VA truly is engaged in this cultural transformation and I’m hopeful that that will continue”. *(Facilitator feedback via listening session)* “Let’s say we all go back and facilitate our groups...and we get the Veterans all fired up and they’re learning, they’re probably going to end up knowing more than a lot of the staff that are working in the VA...and then, when they go back to their clinics, and they hit the biggest wall, then where do you go with that?” (Facilitator feedback via listening session)
	*Available Resources*, or “the level of resources dedicated for implementation and on-going operations, including money, training, education, physical space, and time”.	“I don’t have space in buildings, and then I want to meet where people are so I have to have community MOUs”. (Facilitator feedback via listening session) “At [location] VA our space is tight. I’m sure it’s the same in most VAs”. (Facilitator feedback via listening session)
	*Leadership Engagement*, or the “commitment, involvement, and accountability of leaders and managers with the implementation”.	“I’m looking at it from the programmatic or organizational point of view... without some clear accountability, without giving it importance from the leadership level, how do I pull her as an asset when I need her [to facilitate a group] when she’s supposed to be doing x, y, and z?” (Facilitator feedback via listening session)
	*Relative Priority*, or “individuals’ shared perception of the importance of the implementation within the organization”.	“This needs to be rolled out to staff. We’re rolling this out to Veterans and my greatest fear is we’ve been done all this work with Veterans in nine weeks and I walk into a clinic with staff who have no clue with this is about”. (Facilitator feedback via listening session)
*Characteristics of the Individual*, or the interplay between individuals’ characteristics and their ripple effects through their teams, units, networks, or organizations on implementation	*Other Personal Attributes*, or “a broad construct to include other personal traits such as tolerance of ambiguity, intellectual ability, motivation, values, competence, capacity, and learning style”.	“As a military retiree and career recruiter, my intuition is to get to the “yes”. It was difficult for me at first to not try and help a fellow veteran with advice and my personal perspective. I’ve since learned that I need to trust the process and let them come to their own conclusions and let the group dynamic flow”. (Facilitator feedback via open-ended survey response)
	*Knowledge and Beliefs about the Intervention* refers to “individuals’ attitudes toward the intervention and familiarity with facts or principles related to the intervention”.	“As a Veteran who is recovering from addiction and had PTSD and other health concerns I identified with this on a more personal level, as far as I know what I’ve been through... and I have a good idea at least that this program can help, you know, other Veterans out there just like me and also has the potential to save a lot of lives so that’s where I came from it, that’s what really made me want to come”. (Facilitator feedback via listening session)
	*Self-efficacy* or “individual’s belief in their own capabilities to execute courses of action to achieve implementation goals”.	“I think that I’m certainly walking away with skills, I mean, I feel like I have learned even more skills in facilitating groups, and I do a lot of groups, and so I’m appreciative of those skills”. (Facilitator feedback via listening session) “... it’s an amazing feeling for us as Peer Support Specialists to finally be acknowledged... they’re really implementing this and it makes me so proud”. (Facilitator feedback via listening session)
*Process*, or how the intervention is changed or enacted.	*Planning*, or “the degree to which a scheme or method of behavior and tasks for implementing an intervention are developed in advance, and the quality of those schemes or methods”.	“[In regards to facilitating a challenge experience was], starting groups in other clinics. Logistical arrangements (e.g., setting up clinics, checking stop codes, schedule, making modifications to group to suit needs of specialty programs)”. (Facilitator feedback via open-ended survey response)
	*Engaging*, or “Attracting and involving appropriate individuals in the implementation and use of the intervention through a combined strategy of social marketing, education, role modeling, training, and other similar activities”.	“An avenue for providers to refer patients directly to our group. More education for clinicians. I recruit as much as I can, but I am not a clinician and do not work with veterans in my VA capacity. I work in Environmental Services and that is a full-time job, so I have to squeeze in time to recruit when I can..”. (Facilitator feedback via open-ended survey response) “I found, just stumbled upon some rich ground to sow the seeds is through the...new employee orientations,...as well as employee health, because if I start implementing these concepts into employee health, it just naturally spreads to the Veteran population....getting in the heads of the employees is also key”. (Facilitator feedback via listening session)
	*Executing*, or “carrying out or accomplishing the implementation according to plan”.	“Coming together as facilitators and following a consistent group plan. Meaning with me being a Veteran and my co facilitator being an LPN. It is sometimes hard to stop being a nurse and understanding how veterans think and feel about things”. (Facilitator feedback via open-ended survey response) “…Important in small group dynamics to monitor for one individual “monopolizing” the conversation with specific problem”. (Facilitator feedback via open-ended survey response)

^1^ Descriptions of domains and constructs are taken from the Consolidated Framework for Implementation Research website, https://cfirguide.org/ (accessed on 15 January 2022).

**Table 5:** TCMLH Adaptations and Attendance by Reporting Years, 2016–2018.

TCMLH Duration Adaptations	Adaptation Rationale and Description	Number of TCMLH Groups (%)	Average # of Attendees	Range of Attendees
12-week format	This format was used by one site and involved minor adaptations to the 9-week standard program, and covered all of the same content. The one 12-week program was implemented to accommodate low attendance over the holidays, adding more sessions to cover the content that many had missed when they were unable to attend.	1 (0.75%)	10	10
9-week format (original length)	The 9-week format is the way the TCMLH program was designed to be delivered.	56 (42.11%)	4	1–10
8-week format	The 8-week program was implemented by three sites, because it was a better fit for scheduling needs.	9 (6.77%)	4	1–10
6-week format	Two sites implemented the program over 6 weekly sessions, which POCs explained was a more feasible commitment for Veterans.	49 (36.84%)	7	2–15
5-week format	The 5-week format followed the condensed plan of the 6-week program and was implemented based on scheduling preferences and availability of a facilitator.	5 (3.76%)	5	2–7
4-week format	Two sites implemented 4-week programs. Such programs aimed to cover the core content of the program focusing on the first four sessions of the 9-week program, plus additional content based on needs or interests of the participants.	10 (7.52%)	8	1–15
2-day format	Three programs utilized a 2-day intensive format (e.g., a weekend “retreat”).	3 (2.26%)	13	5–17

**Table 6:** Recruitment Methods Utilized by the VHA System for TCMLH Implementation (n = 8).

Recruitment Methods	No. of Sites Using Method (% of Total Sites)	% Reported that Recruitment Method “Worked Well”	% Reported that Recruitment Method “Did Not Work Well”
Clinician referrals	7 (87.5%)	57.1%	50%
Outreach to veterans by program representatives	7 (87.5%)	85.7%	14.3%
Introduction of program during veteran orientation events	6 (75.0%)	83.3%	16.7%
Passive media advertising (e.g., flyers)	6 (75.0%)	50%	50%
Promotion in other group programs within the VHA system	4 (50.0%)	75%	0%
Follow-up or reminder calls by staff	3 (37.5%)	100%	0%
Word of Mouth	2 (25.0%)	50%	0%

## Data Availability

The deidentified data analyze for the current study are available from the corresponding author upon request.

## Supplementary Materials

The following supporting information can be downloaded at: https://www.mdpi.com/article/10.3390/ijerph19148333/s1, Facilitator Listening Session Guide.
