# Supplementary material for: National Implementation of a Group-Based Program Promoting Patient Engagement and Peer Support in the Veterans Health Administration: A Multi-Methods Evaluation
Source: Int J Environ Res Public Health. 2022 Jul 7;19(14):8333. doi: 10.3390/ijerph19148333 (PMC9321656; doi:10.3390/ijerph19148333)
Supplement: Supplementary file 1 [file ijerph-19-08333-s001.zip › ijerph-1722138-supplementary.pdf]

Facilitator Listening Session Guide

QUESTIONS FOR FACILITATORS POST-TRAINING

Duration: 60 minutes

Introduction: Thank you for agreeing to be a part of this listening session today. You have been asked here today to discuss your overall perceptions about the Whole Health Group Facilitator Training as well as the Whole Health Group Program itself. Our discussion will be recorded in order to ensure we capture all thoughts and perceptions shared by the group. To assist in capturing as much information as possible, please try to speak loudly and clearly and one at a time.

Our discussion is confidential and your responses will not be linked to any identifying information. To that end, I respectfully ask you to keep what is said confidential and in this room. For our evaluation, we will summarize thoughts and perceptions shared today into key themes that we will present to the OPCC&CT in a report which will provide feedback on the facilitator training and program, as well as recommendations for wider dissemination within the VHA. Are there any questions?

Questions & Follow-ups:

1. BRIEFLY → How did you hear about this course?
    a. Follow-up: How were you recruited?
    b. Follow-up: What drew you to the course? What did you find unique about this course description?
2. What overall impact would you say the training has had on you as a Peer Group Facilitator?
    a. Follow-up: What knowledge did you gain or further develop as a result of this training? (e.g., patient-centered care, whole health approaches, PHI, resources within VA or community)
    b. Follow-up: What skills did you gain or further develop as a result of this training? (e.g., facilitator skills, group management skills, accessing resources within VA or community)
    c. Follow-up: What did you think, overall, about the training presentation?
    d. Follow-up: What did you think, overall, about the trainee practice with feedback?
3. Thinking of the training overall, what was missing from your training, and what would you like more of? (e.g., more practice, more feedback, more didactic)
4. What would you say is the value of this Whole Health group program for Veteran health and well-being?
5. What do you see as challenges in implementing this program?
    a. probes: recruitment; 9 weeks; 1 ½ hours
    b. solutions / ideas for what might work better
6. Can you see health outcomes changing for facilitators of the course as well as participants of the course? If so, how?
7. What do you think about integrating mindful awareness activities into peer group programs about self-care with Veterans?
    a. Follow-up: What kind of impact do you think mindful awareness could have on Veterans?
    b. Follow-up: Do you perceive any challenges or barriers with integrating mindful awareness activities in the group sessions?
    c. Are there Veterans for whom mindful awareness will be met with resistance or discomfort; if so, how would you address that?
8. In what ways, if at all, did this training change the way you think about Veteran self-care? (e.g., importance of setting goals, creating action plans, seeking resources, incorporating mindful awareness, and focusing on components of health and well-being etc.)
9. Do you believe that group programs such as this one will help better engage Veterans in healthcare services and resources for their physical and mental well-being?
